# Enabling Health Reform through Regional Health Information Exchange: A Model Study from China

**DOI:** 10.1155/2017/1053403

**Published:** 2017-03-28

**Authors:** Jianbo Lei, Dong Wen, Xingting Zhang, Jiayu Li, Haiying Lan, Qun Meng, Dean F. Sittig

**Affiliations:** ^1^Center for Medical Informatics, Peking University, Beijing, China; ^2^Peking University Third Hospital, Beijing, China; ^3^Health Department of Xinjin County, Chengdu City, Sichuan Province, China; ^4^Center for Statistics and Information, National Health and Family Planning Commission, Beijing, China; ^5^The University of Texas School of Biomedical Informatics, Houston, TX, USA

## Abstract

*Objective*. To investigate and share the major challenges and experiences of building a regional health information exchange system in China in the context of health reform. *Methods*. This study used interviews, focus groups, a field study, and a literature review to collect insights and analyze data. The study examined Xinjin's approach to developing and implementing a health information exchange project, using exchange usage data for analysis. *Results*. Within three years and after spending approximately $2.4 million (15 million RMB), Xinjin County was able to build a complete, unified, and shared information system and many electronic health record components to integrate and manage health resources for 198 health institutions in its jurisdiction, thus becoming a model of regional health information exchange for facilitating health reform. *Discussion*. Costs, benefits, experiences, and lessons were discussed, and the unique characteristics of the Xinjin case and a comparison with US cases were analyzed. *Conclusion*. The Xinjin regional health information exchange system is different from most of the others due to its government-led, government-financed approach. Centralized and coordinated efforts played an important role in its operation. Regional health information exchange systems have been proven critical for meeting the global challenges of health reform.

## 1. Background

The global healthcare community is facing many common challenges, such as the growing burden of care for the elderly, obstacles to public access to medical care, and the uneven distribution of medical resources and healthcare quality. In 1999, the Institute of Medicine (IOM) issued a landmark report, “To Err is Human” [1], citing the need for action to prevent medical errors and improve the quality of healthcare through the use of health information technology (HIT). Since then, HIT has received considerable attention around the world, along with substantial policy and funding support [2]. This interest has increasingly focused on health information exchange (HIE) projects that facilitate the access to and sharing of patient information [3, 4]. Over 280 HIE projects have been launched throughout the US, covering almost every state [5]. However, many questions remain regarding the costs, benefits, feasibility, and financing of such initiatives.

In the US market-oriented approach, HIE collaborations, known as “regional health information organizations” (RHIOs), are extremely varied and generally involve diverse stakeholders joining together to plan, finance, and implement systems for sharing electronic health information [6]. Participants may include hospitals, clinics, laboratories, pharmacies, safety net providers, payers, employers, public health departments, quality improvement organizations, and consumers. In 2009, Harvard's Adler-Milstein et al. surveyed all RHIOs in the US (207) and found that only 55 (27%) were operational, with a limited scope of data exchange focused mainly on laboratory test results [7]. The authors also raised concerns about long-term, self-sustaining financial viability. It is not yet known whether the current US market-oriented approach of offering small grants and waiting to see which RHIOs flourish will work [8].

**Figure 1:**
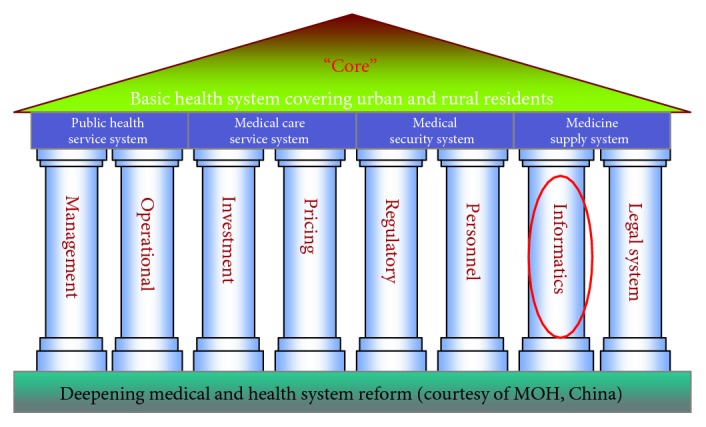
Core foundations (four constructs and eight pillars) of China's health reform.

Amid China's rapid economic development and increasing population, its health system is facing enormous challenges, with accessibility, expensive services, and an uneven quality of care among the most discussed [9]. In 2009, the Chinese government launched the second stage of its ambitious healthcare reform [10]. The overall goal is to create a fair, transparent, practical, and efficient healthcare system. A prominent contribution of this reform is its description of HIT as one of the “four constructs and eight pillars,” or foundations (see Figure 1) of healthcare reform [10].

This statement has brought considerable attention to HITs and to financial investment. It is estimated that the Chinese government invested more in 2010 than it had during the 60 years since 1949, when the People's Republic of China was established. This policy and financial support have enabled the construction of an electronic medical record system, a telemedicine system, and regional health information exchange (RHIE) systems. China's Ministry of Health developed a national health information development strategy for the 12th Five-Year Plan (from 2010 to 2015) called “3521” [11]. This strategy calls for the establishment of three levels (national, provincial, and municipal/regional) of health information exchange platforms; five applications (public health, medical services, a new rural cooperative medical system, an essential drug management system, and integrated patient management); two databases of electronic medical and health records; and a dedicated health network.

Health information technology has generally been considered the main vehicle of the comprehensive reform of China's primary healthcare institutions. Under the “3 Exchange Platforms” of China's “3521” national plan, regional HIE has been used as the starting point for realizing the key health reform goals: safeguarding the public welfare, stimulating patient motivation, and ensuring sustainability through the establishment of mechanisms such as standardized and orderly operations, open and transparent evaluations, comprehensive compensation and investments, and an efficient pharmaceutical supply chain.

Xinjin County in Sichuan province is one of the pilot locations of China's medical information technology reform. In accordance with the conceptualization of urban and rural centralized planning and regional integration, China has used regional health information construction to facilitate healthcare reform, achieving remarkable success. Relatively quickly and economically, Xinjin has turned itself into a successful case study of the revolutionary transformation and integration of an entire information system for all primary healthcare institutions.

## 2. Methods

### 2.1. Setting: Location and Institutional Structures of Xinjin County

**Figure 2:**
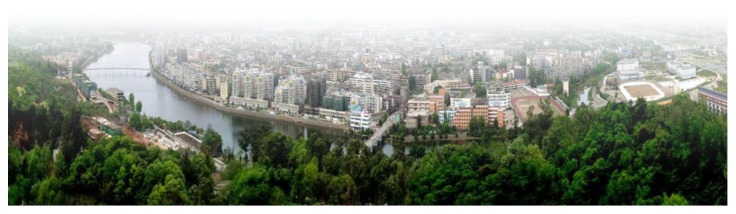
Bird's eye view of Xinjin County.

**Figure 3:**
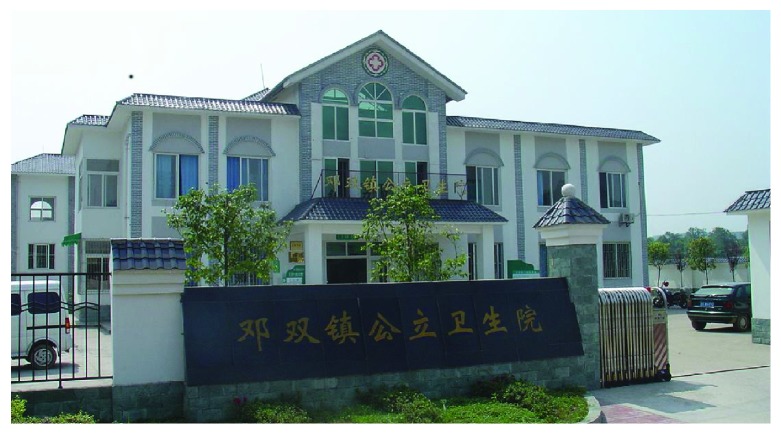
Dengshuang town public health center.

Xinjin County is at the third level of China's four-level (provincial, prefecture, county, and town) administrative hierarchy. One of the 20 county-level administrative units under Chengdu city, the capital of Sichuan province, Xinjin County, is located in the western Sichuan Basin, 28 kilometers south of Chengdu city. The county covers an area of 330 square kilometers and has jurisdiction over 11 towns, one township, and 106 administrative villages (communities), with a total population of 307,800 (see Figures 2 and 3 for Xinjin's geographical location and health institutions).

**Figure 4:**
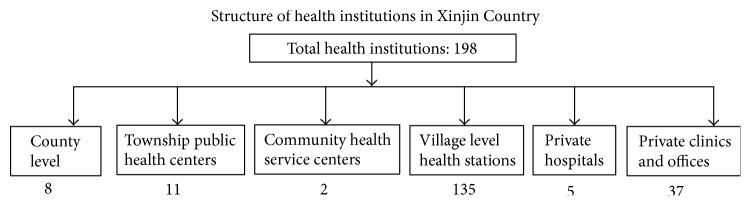
Structure of health institutions in Xinjin County.

**Figure 5:**
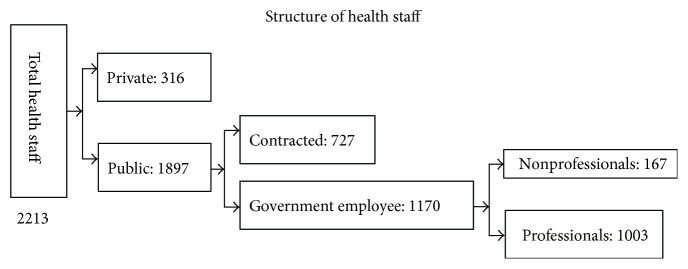
Structure of health staff in Xinjin County.

**Figure 6:**
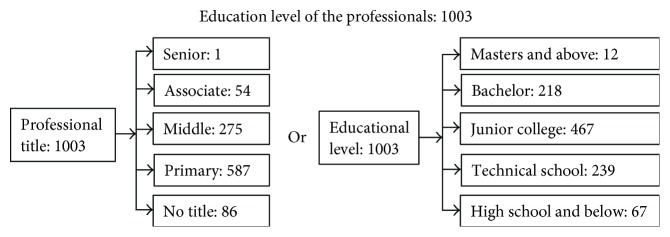
Structure of education level of health staff in Xinjin County.

Xinjin County has 198 types of medical and health institution, including eight medical and health institutions at the county level, 11 township public health centers, two community health service centers, 135 village-level medical and health institutions, five private hospitals, and 37 clinics and offices. These facilities employ a total of 2213 medical staff, many of whom lack advanced computer skills (see Figures 4–6 for the structure of Xinjin's health institutions and medical staff).

Xinjin was ranked among the top 25 of China's 100 most investment-worthy middle-to-small cities, 30th among western China's top 100 counties, and 11th in terms of county-level comprehensive economic power in Sichuan province. Xinjin was chosen as one of the first trial counties for national essential drug management system implementation in Sichuan province, as part of the medical and health system reform in Chengdu city.

### 2.2. Rationale of and Challenges to Reform

As a healthcare reform trial county, Xinjin is required to conduct experiments on how to achieve the general reform goal of an equal distribution of public health services and consistent quality of basic healthcare. Specifically, Xinjin must implement a standardized and normalized primary care-based system, as it is an established county-level city. Its major aims in this trial include building an advanced health service system at the county, township, and village levels; improving the quality and level of health services; and promoting the development of unified urban and rural health quality. Though Xinjin has made progress, a bottleneck persists with large discrepancies in terms of size, staff competency, and resources across its 198 health institutions. Moreover, the health institutions feature uneven, heterogeneous information technology capabilities. The lack of a unified management, monitoring system, and information sharing mechanism will make it impossible to achieve the goal of building a unified, efficient, interoperable, and shared healthcare delivery system.

Xinjin has been facing several major difficulties. The healthcare competency of its 1003 professional health staff members is poor. Less than 25% (230) have formal college education. The 198 institutions have financial, technical, and human resources of varying quality. For example, healthcare providers are unable to share patient information and administrative information across the institutions using traditional paper health records or fragmentary information systems. In addition, the level of health services and insurance coverage cannot be monitored, let alone improved. Care quality and service levels across the institutions are uneven, and the institutions are complete silos in terms of both clinical and administrative information. Meanwhile, public needs and complaints are increasing. To resolve these issues, Xinjin County started to build an RHIE with a unified, shared information system for all 198 institutions under the aegis of national health reform. An information system was needed to normalize and standardize the distribution of healthcare, to monitor and improve care quality, and to enhance the capacity of health services. Regional health information exchanges have been considered facilitators in coordinating and deepening the medical and health system reform and ultimately improving public health.

### 2.3. General Objectives

**Figure 7:**
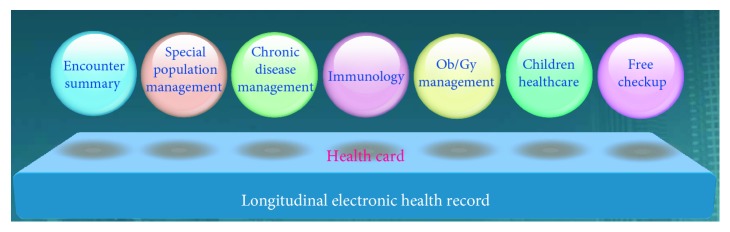
Technical architecture of the regional HIE system.

As a foundational part of the municipal/regional health exchange platform, the Xinjin regional HIE plan, the lowest level of the national three-level information exchange platform, has been framed as the “1131” network (one county-level data center, one health private network, three application systems [medical, public health, and county-level management platform], and one resident health card). The Xinjin regional HIE system will take an existing electronic health record (EHR) as the core and a health insurance/health card as the link to develop a shared exchange platform as the basis for the integration of electronic medical records (EMRs), drug administration, public health, two-way referral, and teleconsultation, while aiming to build a unified, efficient, effective, unblocked, and safe regional health information network. The technical architecture of the HIE system is illustrated in Figure 7.

Ultimately, each of Xinjin's 307,800 residents will receive a unique health card, a smart card issued whenever the resident first accesses health services at any health institution, enabling an unimpeded flow of information when any institution within Xinjin is visited. Patients will be able to make an appointment, register to see a doctor, and pay for their healthcare, and their clinician will be able to order medication using the card. Information on each patient-doctor encounter will be linked to other data in the county data center. Every doctor across the 198 institutions will be able to view complete health records for all patients and receive decision support from the regional HIE system throughout the healthcare process. Health administrators with the proper authority will monitor and manage the drug administration, quantity, and quality of the health services provided every day, or every month, across the whole county in real time.

### 2.4. Development and Implementation

As no truly successful case of a complete regional HIE system in China exists, we selected a vendor based on three major criteria. First, the vendor needed a proven record of successfully implementing hospital information systems of various sizes; second, the vendor needed successful experience with similar regional health information projects; third, the vendor needed a team of dedicated professionals who could work onsite to develop and install the systems with the help of local staff with clinical knowledge and health management experience. Through a formal open bidding process, an expert committee consisting of policy makers, informatics professionals, physicians, nurses, and health administrators chose a vendor who had developed a complete proposal for the regional HIE systems suitable for our region and our requirements based on the above three major decision factors. During the implementation period, we created a three-stage plan, in which each stage has its own goals and acceptance criteria:

Stage one (July 2008–April 2009):

- Build a preliminary data center, data exchange platform, and overall architecture of the HIE system.
- Concurrently, develop related health information systems, such as systems for urban and rural health management, medical management, pharmaceutical management, chronic disease management, community health, maternal and child health, mental health, infectious disease management, endemic disease management, health education, health supervision, health emergency, electronic prescribing, asset finance, and personnel management.
- Employ them in county and township public health agencies as well as rural community health service stations.

Stage two (June 2009–May 2010):

- Improve the regional data center and county-level data management platform.
- Concurrently, develop and employ core systems of the HIE project, such as the health card management system, EMR system, and patient or financial management systems.
- Implement interfaces between systems and deploy all of the stage-one and stage-two systems at all major health institutions.

Stage three (June 2010-2011):

- Continue to improve existing system functions by strengthening the data center and county-level management platform.
- Develop and construct systems for drug logistics and distribution, resident self-checkup, performance appraisal, regional digital laboratory test and imaging, medical quality management, hospital queue and calling, and health checkup, as well as the patient portal.
- Extend the upgraded regional health information system to the private hospitals and village health stations to achieve the final goal of integrating the management of the county's urban and rural medical and health institutions.

## 3. Results

Xinjin County began implementing its regional HIE project in 2008. After the three stages of construction, Xinjin County has built a preliminary regional information system covering the county health bureau and seven county-level medical institutions, 11 village and town public hospitals, two community health service centers, and more than 60 new rural community health service stations, including regional laboratory service and imaging system centers across seven medical institutions.

The operational HIE system consists of a fast, efficient, smooth, and safe regional health information network that takes residents' electronic health records as the center and provides a platform for sharing and exchanging as the base. The major module functionalities include health card management, electronic medical records, computer-based provider order entry (CPOE), regional imaging, laboratory services, drug supply, performance assessment, maternal and child healthcare, health examination, self-checkup services, disease surveillance, chronic disease management, health insurance exchange, administrative management, and e-government.

Patient information was collected at each health institution electronically through various modules and transferred to a county-level regional health exchange data center using a special internal health private network. The workflow of each health institution was digitalized and partially optimized. Important health information such as billing and administrative data can be shared among different institutions. Patients can easily create their own health information archives, including an immunization registry and logs for health checkups, doctor visits, billing, and health information queries using a single card.

The growth and usage of the Xinjin County HIE systems have been stable since June 2012 (see Table 1). No significant increases in transactions or uploads that could bring the system down have occurred.

Within three years, Xinjin built a systematic network of services that realized, first, the digitalization of basic health services, then the electronic monitoring and management of the entire health process, and, finally, the centralized exchange of patient and administrative data to assist in accessing, sharing, monitoring, and analyzing health data and health services. The Xinjin regional HIE project is not just an exchange platform but also contains various operating information systems within each of its 198 institutions.

The implementation of the Xinjin HIE project has clearly improved health services and management, while significantly advancing the reform of China's medical and health system.

## 4. Discussion

### 4.1. Investment and Unique Organizational Model

During the first and second phases of the project, 6.33 million RMB ($1.02 million USD) was spent, consisting of 1.23 million RMB ($200,000 USD) on software, 4.65 million RMB ($750,000 USD) on hardware, and 0.45 million RMB ($70,000 USD) on network and other operating costs. Building the county data center costs 1.07 million RMB ($170,000 USD). A total of 8.47 million RMB ($1.36 million USD) has been invested in the third phase, comprising 1.28 million RMB ($210,000 USD) on software, 6.15 million RMB ($990,000 USD) on hardware, and 1.04 million RMB ($170,000 USD) on network and other operating costs. An additional 0.77 million RMB ($120.000 USD) has been reallocated to the data center. All of these investments were made by the local government. Further investment is inevitable, as this is a government-led project. The costs of RHIOs in the US are usually divided into three phases: planning, development and implementation, and operations [6]. Investments in the Xinjin regional HIE are smaller than regular investments in US RHIOs (see Table 2 for a comparison), and further funding should be easy to obtain.

The major difference is that the cost in the planning phase was zero for Xinjin since this was the responsibility of the government, and no external costs were incurred in this phase. In China's unique healthcare system, most of the health institutions/physicians are owned and financed by the government. For instance, 161 of Xinjin's 198 health institutions are public. Therefore, in China's regional HIE project, no complex stakeholders or public-private RHIOs were required. The government is both the owner and user of the HIE project since it owns most of the health institutions, and the users of the HIE system are government employees. The government is also the major stakeholder. The strongest driver of the regional HIE is the public good—in this case, the national health reform.

### 4.2. Achievements Related to Health Reform

#### 4.2.1. Benefits to the Public: Two Major Problems Addressed

The two major problems the China health reform is intended to solve—difficulties in accessing healthcare and the high costs of doctor visits—have been resolved. Residents of Xinjin County now enjoy more standardized, accurate, simple, and convenient health services because of the digitalized health service process. Residents enjoy better health services provided by upper-level hospitals through telemedicine and regional laboratory and image centers, while paying according to their village station level. Ambulatory and inpatient costs can be reimbursed electronically in real time. Personal health information, including encounter histories, prescriptions, medications, and laboratory tests, are accessible through a patient portal in the electronic health record system. The quality of laboratory tests is monitored by a centralized laboratory service system, and laboratory results are sharable, greatly reducing redundant tests. Therefore, residents have begun to enjoy more convenient, equalized, high-quality, and low-cost health services.

#### 4.2.2. Benefits to Medical Staff: Improved Quality of Care and Health Services

Further standardizing service processes and the clinical behavior of the medical staff have improved the quality of care. The unequal knowledge skills of medical staff across the county are addressed through online training and online knowledge databases such as the most recent diagnostic and treatment knowledge or online technical support provided by senior doctors. Service levels and health service efficiency have been improved via electronic documentation, information sharing, and the prevention of data entry duplication. Staff members' workloads and performance can be measured and evaluated to foster enthusiasm among the medical staff.

#### 4.2.3. Benefits to Health Departments: Improved Health Agency Management and Operational Capability

First, through the EHR management and reporting systems, health departments can monitor the real-time health status of residents in the area, determine a disease spectrum, identify causes of death and health hazards, and develop interventions targeted to health promotion, thus improving health management at the appropriate decision-making level and the health of all residents. Second, through the drug supply chain and prescription statistics, the system-level health department can monitor the proportion of drug use, abnormally large prescriptions, or the inappropriate use of antibiotics to promote a rational use of drugs and reduce medication costs; these were significant issues before the health reform began. Third, through comprehensive data exchange and analysis, health administration departments can now keep track of the working status and health service satisfaction of county health institutions, allowing them to identify problems and find solutions faster. Finally, data sharing and centralized data analysis enable administrators to fully grasp the conditions and usage status of health resources, help to optimize the configuration of health resources, and improve efficiency in the use of government funds.

### 4.3. Lessons Learned

#### 4.3.1. Strong Executive Support and Efficient Coordination from All Departments

Strong leadership support has been frequently cited as the primary success factor for a complex health IT project, especially a regional HIE [13]. Leadership support is relatively easy to obtain in China's cultural and political context. Under the national health reform strategy, Xinjin County's government attached great importance to the health information exchange project. Major leaders were personally involved in project decision making, and the project was made part of the county's high-priority work, annual strategy, and tangible work plans. A mechanism for coordination between two new working groups, a leadership team and working team, was established specifically for this project. The project leadership team comprised county-level leaders who were heads of the health and other relevant departments of the county government, such as the County Strategy Planning Bureau, Social and Health Insurance Bureau, and Finance Bureau; these guided, coordinated, and supervised the project. Another working group comprising health management, IT professionals, and medical staff was established to manage the execution of the project.

#### 4.3.2. Challenge of the Great Variation in Computer and Clinical Skills within the County: Propaganda, Mobilization, and Training

One of the key challenges in implementing a high-level, technology-oriented health information project starting from grassroots-based health institutions was the uneven health resources across institutions—especially the uneven level of clinical and computer skills among healthcare staff in underdeveloped areas of the county. About one-fifth of the health staff in Xinjin County had limited computer skills. The implementation team also faced enormous resistance from staff members. The project working group thus attached great importance to propaganda, mobilization, and training. A series of comprehensive tactics were applied:

- First, mobilization meetings and seminars were held to change mindsets and identify and solve problems.
- Second, training was offered in batches, including for computer operation and software functionality, to improve the application level.
- Third, the training of network administrators was reinforced through intensive sessions and weekly meetings, and employment licenses were evaluated, thus constantly improving the capacity of the daily maintenance of the computers, software, and network of the network management personnel.
- Fourth, competency training for senior work staff was strengthened. One effective way was facilitating onsite observation, learning, and exchanges, which eliminated the fear of using computers among older workers; also, designated personnel were assigned to help senior staff members solve their problems; addressing staff members' fears was especially important during the early period.

These efforts led to most health personnel being able to operate the computers and information systems. The health information technology application level has been significantly improved. The determination and support of the working team produced very positive results.

#### 4.3.3. Design Strategies: Systematic and Centralized Planning and Unified Information System, Ensuring Interoperability and Sharing

The strong executive support enabled unified and centralized planning. The members of the working team investigated all requirements from various types of health-related staff and worked with the vendor to develop a unified infrastructure for the HIE project and subsystems. National and ministerial standards must be developed and applied to ensure interoperability and sharing. All primary, secondary, and tertiary institutions must connect to the county's unified network, and all required modules and subsystems must be provided and supported by the working and implementation team. This approach of centralized planning and unified development, deployment, and support greatly reduced costs, improved efficiency, and prevented the formation of islands of information.

#### 4.3.4. Implementation Strategies: Refining and Decomposing Targets, Step-by-Step Implementation with Regular Supervision and Report

Methodologically, the regional HIE is a huge, complex project of system engineering. The working team decomposed the overall goals, refined them into specific phase-based aims and actions, and then assigned them to responsible entities and personnel along with quality and time requirements. Weekly progress reports and analyses were made. If there were problems, the manner and timing of follow-up actions were defined and later measured. Strong executive leadership support and effective strategies ensured the quality and time requirements for building these complex regional HIE systems.

### 4.4. Challenges and Lessons Learned

#### 4.4.1. Integration Difficulties with Many Heterogeneous Systems

Health services at the county level comprise primary care, secondary care, and public services. Therefore, many special applications and modules are found across various health institutions, such as maternal and child health modules, a planned immunization module, a health surveillance system, a disease control system, insurance module, and billing module. Integration and interoperability are always a challenge for any regional or cross-departmental information system. We have partially solved this problem through unified planning and executive leadership by replacing most of the old systems with new, unified systems. This approach was feasible for Xinjin, where basic information systems were weak, as the working team was given strong executive support. Complicating the situation, however, is that various upper levels of China's public health system such as national, provincial, and municipal information systems are in their initial construction phases. Docking and integrating county information into upper-level regional information systems to ensure a wider scope of health information sharing is proven problematic. This is especially true for public health reporting and surveillance systems. A variety of data element, data exchange, interface, and even practice standards should be investigated, developed, and enforced by the central government.

#### 4.4.2. Shortage of Professionals Greatly Affecting HIT Usage Level

The county suffers from a severe lack of interdisciplinary talent, as regional health information construction is still in its infancy in China. Very few mature and successful experiences are available to learn from. People need to understand health information technologies, the reality of health work, the nature of health management, and how to combine the above knowledge to generate constructive suggestions and ensure a smooth HIT project path with few detours. China has very few such interdisciplinary education programs. Therefore, Xinjin County, a grassroots county agency, is naturally experiencing an extreme shortage of interdisciplinary talent. Moreover, the county lacks professional computing talent. Several medical and health institutions lack professional system maintenance personnel. An incredible reality in Xinjin is that the role of network manager might be served by a doctor or accounting officer rather than an IT professional; this leaves many problems unresolved.

### 4.5. Next Plan

The regional HIE project has achieved its preliminary goals. Next, we will pursue the digitalization and centralization of several special health services, such as testing and reading laboratory, electrocardiography (EKG), and imaging results, which require higher-quality clinical skills. After unified and centralized laboratory, EKG, and imaging systems are implemented, the grassroots institutions will mainly be responsible for collecting laboratory specimens, taking images, and conducting EKG exams, while central and high-quality county-level institutions will handle the testing of laboratory specimens, the reading and reporting of images and EKG results, and related teleconsultation services. Meanwhile, we plan to apply wireless and RFID technology to ensure mobile and telecare and reduce medical errors. Finally, one challenging task in the pipeline is to evaluate how well the regional HIE project has been used and what health outcomes it has achieved.

## 5. Conclusion

An RHIE is an important vehicle for achieving the health reform policy goals of providing safe, effective, convenient, and inexpensive medical and health services. However, there are very few successful RHIE cases because of the cultural, organizational, political, systematic, and technological challenges involved. Further quantitative analyses are needed to evaluate the outcomes of such projects. However, this case study shows that a government-led, non-market-oriented, organization-wide stakeholder approach can be used to implement an RHIE project relatively quickly and with modest funding and that an RHIE helps promote health reform goals. In achieving some of the goals defined by China's second health reform, we have also accumulated experience and lessons that we hope will be valuable to others. China has formally begun the large-scale national construction of hospitals and RHIE projects. We trust that China's experiences will continue to contribute to the global community.

## Figures and Tables

**Table 1:** Usage of regional health information exchange system of Xinjin County.

	June 2012			March 2014			
Systems	Number of users	Total size of data (MB)	Increase per day (MB)	Number of users	Total size of data (MB)	Increase per day (MB)	Number of transactions
Outpatient doctor's station	652	12,645	17.6	662	27,904	40.3	4582
Inpatient doctor's station	652	22,694	10.1	675	46,478	43.5	1352
Out/ED billing station	30	9324	11.1	44	18.514	15.4	3458
Inpatient billing station	45	38,797	64.7	48	72,469	82.35	155
Nurse station	766	12,834	18.0	796	30.487	20.5	
Regional imaging sharing	62	10 TB	8 GB	67	20 TB	19 GB	272
Regional lab sharing	68	3.65 GB	22.4	69	10.25 GB	52.2	300/36
Regional EKG sharing	59	6.38	0.11	61	102	0.9	39
Regional quality control system	23	576.2	9.43	28	1088	12	56
Regional public health management		350 GB	10 GB	56	539 GB	52 GB	
Rational use of drugs		9130	0.03		12,500	3.5	5634
Electronic health records	Unknown (352,561 records in total)	14,711	8.23	1512	23,815	10.6	Unknown

**Table 2:** Comparison of costs in the three RHIO phases between the US [12] and Xinjin.

Stage of development	Costs in US	Costs in Xinjin regional HIE
Planning	$300,000 to $1 million	0
Development and implementation	$3 million to $10 million	$2.38 M (14.8 million RMB)
Operations	$2 million to $5 million	$0.16 M (100 M RMB)

## Abbreviations

EHR: Electronic health record
EKG: Electrocardiography
EMR: Electronic medical record
HIE: Health information exchange
HIT: Health information technology
IOM: Institute of Medicine
RHIE: Regional health information exchange
RHIO: Regional health information organization.

## Additional Points

*Availability of Data and Material*. All relevant data are within the paper and its supporting information files.
